# 4-Meth­oxy­benzamidinium 2,6-dimeth­oxy­benzoate

**DOI:** 10.1107/S160053681105519X

**Published:** 2012-01-07

**Authors:** Gustavo Portalone

**Affiliations:** a Chemistry Department, "Sapienza" University of Rome, P.le A. Moro, 5, I-00185 Rome, Italy

## Abstract

The title compound, C_8_H_11_N_2_O^+^·C_9_H_9_O_4_^−^, was synthesized by the reaction of 4-meth­oxy­benzamidine (4-amidino­anisole) and 2,6-dimeth­oxy­benzoic acid. The structure consists of non-planar pairs of hydrogen-bonded 4-meth­oxy­benzamidinium cations and 2,6-dimeth­oxy­benzoate anions. In the cation, the amidinium group is tilted by 27.94 (10)° with respect to the benzene ring. In the anion, the sterically bulky *ortho*-meth­oxy substituents force the carb­oxy­ate group to be twisted away from the plane of the benzene ring by 73.24 (6)°. The ions are further associated in the crystal into chains along the *b*-axis direction by inter­molecular N—H⋯O hydrogen bonds.

## Related literature

For the biological and pharmacological relevance of benzamidine, see: Marquart *et al.* (1983 ▶); Sprang *et al.* (1987 ▶); Bode *et al.* (1990 ▶); Powers & Harper (1999 ▶); Grzesiak *et al.* (2000 ▶). For the structure of benzamidine, see: Barker *et al.* (1996 ▶). For supra­molecular association in proton-transfer adducts containing benzamidinium cations, see; Papoutsakis *et al.* (1999 ▶); Portalone (2008 ▶, 2010 ▶). For the structure of benzdiamidine, see: Jokić *et al.* (2001) ▶. For the ortho­rhom­bic and tetra­gonal polymorphs of 2,6-dimeth­oxy­benzoic acid, see: Swaminathan *et al.* (1976 ▶); Bryan & White (1982 ▶); Portalone (2009 ▶, 2011 ▶). For the analysis of benzene ring deformations induced by substitution, see: Schultz *et al.* (1993 ▶); Portalone *et al.* (1998 ▶); For computation of ring patterns formed by hydrogen bonds in crystal structures, see: Etter *et al.* (1990 ▶); Bernstein *et al.* (1995 ▶); Motherwell *et al.* (1999 ▶). 
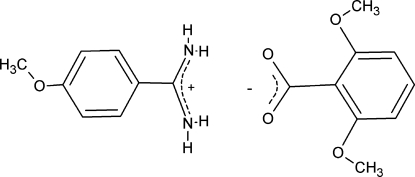


## Experimental

### 

#### Crystal data

C_8_H_11_N_2_O^+^·C_9_H_9_O_4_^−^
*M* *_r_* = 332.35
Monoclinic, 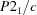

*a* = 12.6594 (3) Å
*b* = 9.6754 (2) Å
*c* = 13.7923 (4) Å
β = 99.241 (2)°
*V* = 1667.42 (7) Å^3^
*Z* = 4
Mo *K*α radiation
μ = 0.10 mm^−1^
*T* = 298 K
0.30 × 0.30 × 0.25 mm

#### Data collection

Oxford Diffraction Xcalibur S CCD diffractometer
Absorption correction: multi-scan (*CrysAlis RED*; Oxford Diffraction, 2006 ▶) *T*_min_ = 0.971, *T*_max_ = 0.976
76316 measured reflections
4217 independent reflections
3914 reflections with *I* > 2σ(*I*)
*R*_int_ = 0.026

#### Refinement

*R*[*F* ^2^ > 2σ(*F* ^2^)] = 0.060
*wR*(*F* ^2^) = 0.131
*S* = 1.17
4217 reflections
239 parameters
H atoms treated by a mixture of independent and constrained refinement
Δρ_max_ = 0.32 e Å^−3^
Δρ_min_ = −0.18 e Å^−3^

### 

Data collection: *CrysAlis CCD* (Oxford Diffraction, 2006 ▶); cell refinement: *CrysAlis RED* (Oxford Diffraction, 2006 ▶); data reduction: *CrysAlis RED*; program(s) used to solve structure: *SIR97* (Altomare *et al.*, 1999 ▶); program(s) used to refine structure: *SHELXL97* (Sheldrick, 2008 ▶); molecular graphics: *WinGX* (Farrugia, 1997 ▶); software used to prepare material for publication: *WinGX* (Farrugia, 1999 ▶).

## Supplementary Material

Crystal structure: contains datablock(s) I, global. DOI: 10.1107/S160053681105519X/rz2691sup1.cif

Structure factors: contains datablock(s) I. DOI: 10.1107/S160053681105519X/rz2691Isup2.hkl

Supplementary material file. DOI: 10.1107/S160053681105519X/rz2691Isup3.cml

Additional supplementary materials:  crystallographic information; 3D view; checkCIF report

## Figures and Tables

**Table 1:** Hydrogen-bond geometry (Å, °)

*D*—H⋯*A*	*D*—H	H⋯*A*	*D*⋯*A*	*D*—H⋯*A*
N1—H1*A*⋯O1	0.93 (2)	1.84 (2)	2.7576 (18)	169 (2)
N1—H1*B*⋯O2^i^	0.88 (2)	1.91 (2)	2.7166 (18)	152 (2)
N2—H2*B*⋯O1^ii^	0.86 (3)	2.31 (2)	2.868 (2)	123 (2)
N2—H2*A*⋯O2	0.91 (3)	2.08 (3)	2.976 (2)	170 (2)

Symmetry codes: (i) 
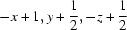
; (ii) 
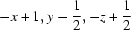
.

**Fig. 1.**
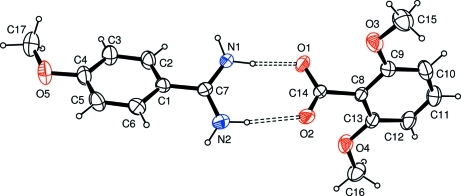
The asymmetric unit of the title compound, showing displacements ellipsoids drawn the 50% probability level. H atoms are shown as small spheres of arbitrary radii.

**Fig. 2.**
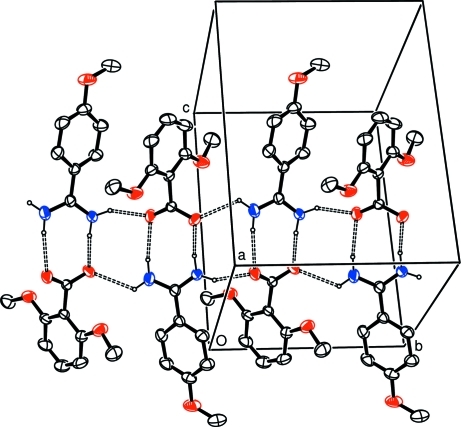
Crystal packing diagram for the title compound. Displacements ellipsoids are at the 50% probability level. H atoms are shown as small spheres of arbitrary radii. For the sake of clarity, H atoms not involved in hydrogen bonding (dashed lines) have been omitted.

## supplementary crystallographic information

### Comment

This Laboratory is currently engaged in the systematic structural analysis of molecular salts of benzamidine (Portalone, 2008, 2010). Benzamidine is a strong Lewis base and its cation can be easily anchored onto numerous inorganic and organic anions and polyanions, largely because of the presence of four potential donor sites for hydrogen-bonding. Consequently, benzamidinium ions appear to be very promising building blocks in supramolecular chemistry as multiple hydrogen bonding donor. Benzamidine has biological and pharmacological relevance (Powers & Harper, 1999; Grzesiak *et al.*, 2000). Its cation has also been included in a number of protein structures (Marquart *et al.*, 1983; Sprang *et al.*, 1987; Bode *et al.*, 1990).

The asymmetric unit of the title compound comprises a non-planar R^2^_2_(8) hydrogen-bonded pair formed by one 4-methoxybenzamidinium cation and one 2,6-dimethoxybenzoate anion (Fig. 1). In the anion, the *o*-methoxy substituents force the carboxy group to be twisted away from the plane of the phenyl ring by 73.24 (6)°. The C—O distances of the carboxylate group range from 1.2440 (18) to 1.2509 (18) Å, indicating the delocalization of the negative charge. The pattern of bond lengths and bond angles of the phenyl ring is consistent with that reported in the structure determination of the two polymorphs of 2,6-dimethoxybenzoic acid (Swaminathan *et al.*, 1976; Bryan & White, 1982; Portalone, 2009, 2011), and a comparison of the present results with those obtained for similar benzene derivatives in the gas phase (Schultz *et al.*, 1993; Portalone *et al.*, 1998) shows no appreciable effects of the crystal environment on the ring deformation induced by substituents. In the cation, the amidinium group forms a dihedral angle of 27.94 (10)° with the phenyl ring, which is close to the values observed in neutral benzamidine (22.8°, Barker *et al.*, 1996) as well as in protonated benzamidinium (20.7°, Papoutsakis *et al.*, 1999; 28.5 and 31.9°, Portalone, 2008, 2010) and benzdiamidine (24.5°, Jokić *et al.*, 2001). The lack of planarity in all these systems is obviously caused by steric hindrances between the H atoms of the aromatic ring and the amidine moiety. The pattern of bond lengths and bond angles of the benzamidinium cation agrees with that reported in previous structural investigations (Papoutsakis *et al.*, 1999; Portalone, 2008, 2010). In particular the amidinium group, true to one's expectations, features similar C-N bonds [1.299 (2) and 1.316 (2) Å], evidencing the delocalization of the π electrons and double-bond character.

Analysis of the crystal packing (Fig. 2) shows that the pairs are associated in the crystal by extensive hydrogen bonding. Those pairs of cations and anions are joined by intermolecular R^4^_4_(8) N—H···O interactions (Etter *et al.*, 1990; Bernstein *et al.*, 1995; Motherwell *et al.*, 1999) (Table 1) leading to a chain structure running along the *b* direction.

### Experimental

The title compound was formed during cocrystallization in a 1:1 molar ratio of 2,6-dimethoxybenzoic acid (1 mmol, Aldrich at 98% purity) and 4-methoxybenzamidine (1 mmol, Fluka at 96% purity). The two components were dissolved in water (10 ml) and gently heated under reflux for 3 h. After cooling the solution to an ambient temperature, colourless crystals suitable for single-crystal X-ray diffraction were grown by slow evaporation of the solvent after two weeks.

### Refinement

The amine H atoms were located in a difference Fourier map and refined freely. All other H atoms could be identified in difference Fourier maps, but were placed in calculated positions, with C—H = 0.97 Å (phenyl) and 0.99 Å (methyl), and refined as riding on their carrier atoms. The *U*_iso_ values were kept equal to 1.2*U*_eq_(C) or 1.5*U*_eq_(C) for methyl H atoms.

### Crystal data

**Table:**

C_8_H_11_N_2_O^+^·C_9_H_9_O_4_^−^	*F*(000) = 704
*M**_r_* = 332.35	*D*_x_ = 1.324 Mg m^−^^3^
Monoclinic, *P*2_1_/*c*	Mo *K*α radiation, λ = 0.71069 Å
Hall symbol: -P 2ybc	Cell parameters from 41031 reflections
*a* = 12.6594 (3) Å	θ = 2.9–34.8°
*b* = 9.6754 (2) Å	µ = 0.10 mm^−^^1^
*c* = 13.7923 (4) Å	*T* = 298 K
β = 99.241 (2)°	Tablets, colourless
*V* = 1667.42 (7) Å^3^	0.30 × 0.30 × 0.25 mm
*Z* = 4	

### Data collection

**Table:**

Oxford Diffraction Xcalibur S CCD diffractometer	4217 independent reflections
Radiation source: Enhance (Mo) X-ray source	3914 reflections with *I* > 2σ(*I*)
graphite	*R*_int_ = 0.026
Detector resolution: 16.0696 pixels mm^-1^	θ_max_ = 28.5°, θ_min_ = 2.9°
ω and φ scans	*h* = −16→16
Absorption correction: multi-scan (*CrysAlis RED*; Oxford Diffraction, 2006)	*k* = −12→12
*T*_min_ = 0.971, *T*_max_ = 0.976	*l* = −18→18
76316 measured reflections	

### Refinement

**Table:**

Refinement on *F*^2^	Primary atom site location: structure-invariant direct methods
Least-squares matrix: full	Secondary atom site location: difference Fourier map
*R*[*F*^2^ > 2σ(*F*^2^)] = 0.060	Hydrogen site location: inferred from neighbouring sites
*wR*(*F*^2^) = 0.131	H atoms treated by a mixture of independent and constrained refinement
*S* = 1.17	*w* = 1/[σ^2^(*F*_o_^2^) + (0.0405*P*)^2^ + 0.878*P*] where *P* = (*F*_o_^2^ + 2*F*_c_^2^)/3
4217 reflections	(Δ/σ)_max_ < 0.001
239 parameters	Δρ_max_ = 0.32 e Å^−^^3^
0 restraints	Δρ_min_ = −0.18 e Å^−^^3^

### Special details

**Table:**

Experimental. CrysAlis RED, Oxford Diffraction Ltd., Version 1.171.32.29 (release 10-06-2008 CrysAlis171 .NET) (compiled Jun 10 2008,16:49:55) Empirical absorption correction using spherical harmonics, implemented in SCALE3 ABSPACK scaling algorithm.
Geometry. All s.u.'s (except the s.u. in the dihedral angle between two l.s. planes) are estimated using the full covariance matrix. The cell s.u.'s are taken into account individually in the estimation of s.u.'s in distances, angles and torsion angles; correlations between s.u.'s in cell parameters are only used when they are defined by crystal symmetry. An approximate (isotropic) treatment of cell s.u.'s is used for estimating s.u.'s involving l.s. planes.
Refinement. Refinement of F^2^ against ALL reflections. The weighted R-factor wR and goodness of fit S are based on F^2^, conventional R-factors R are based on F, with F set to zero for negative F^2^. The threshold expression of F^2^ > 2σ(F^2^) is used only for calculating R-factors(gt) etc. and is not relevant to the choice of reflections for refinement. R-factors based on F^2^ are statistically about twice as large as those based on F, and R- factors based on ALL data will be even larger.

### Fractional atomic coordinates and isotropic or equivalent isotropic displacement parameters (Å^2^)

**Table:**

	*x*	*y*	*z*	*U*_iso_*/*U*_eq_	
O5	0.10799 (10)	−0.11594 (17)	−0.24016 (10)	0.0540 (4)	
N1	0.44446 (13)	−0.09706 (15)	0.15597 (11)	0.0413 (4)	
H1A	0.4973 (17)	−0.110 (2)	0.2099 (16)	0.049 (6)*	
H1B	0.4294 (17)	−0.011 (2)	0.1385 (15)	0.045 (5)*	
N2	0.39964 (14)	−0.32294 (16)	0.15666 (14)	0.0467 (4)	
H2A	0.442 (2)	−0.329 (3)	0.2161 (19)	0.063 (7)*	
H2B	0.3761 (19)	−0.396 (3)	0.1250 (18)	0.059 (7)*	
C1	0.31657 (12)	−0.17948 (16)	0.02049 (11)	0.0303 (3)	
C2	0.33754 (13)	−0.07531 (19)	−0.04288 (12)	0.0376 (4)	
H2	0.4006	−0.0181	−0.0250	0.045*	
C3	0.27065 (13)	−0.0509 (2)	−0.13109 (13)	0.0408 (4)	
H3	0.2871	0.0216	−0.1749	0.049*	
C4	0.17991 (13)	−0.13163 (19)	−0.15559 (12)	0.0375 (4)	
C5	0.15748 (13)	−0.2361 (2)	−0.09328 (14)	0.0426 (4)	
H5	0.0939	−0.2924	−0.1109	0.051*	
C6	0.22544 (13)	−0.26050 (18)	−0.00599 (13)	0.0388 (4)	
H6	0.2096	−0.3344	0.0370	0.047*	
C7	0.38977 (12)	−0.20070 (15)	0.11435 (12)	0.0316 (3)	
C17	0.12111 (18)	0.0003 (3)	−0.30073 (15)	0.0591 (6)	
H17A	0.1146 (14)	0.0864 (14)	−0.2636 (7)	0.089*	
H17B	0.0653 (12)	−0.0014 (10)	−0.3597 (11)	0.089*	
H17C	0.1925 (12)	−0.0034 (10)	−0.3209 (11)	0.089*	
O1	0.61718 (10)	−0.11290 (12)	0.30539 (9)	0.0400 (3)	
O2	0.55414 (10)	−0.31629 (12)	0.34359 (10)	0.0421 (3)	
O3	0.63894 (11)	−0.06691 (15)	0.52149 (10)	0.0517 (4)	
O4	0.78817 (10)	−0.40567 (14)	0.34411 (9)	0.0447 (3)	
C8	0.71797 (11)	−0.23514 (15)	0.43547 (11)	0.0276 (3)	
C9	0.72422 (14)	−0.15283 (17)	0.51889 (12)	0.0366 (4)	
C10	0.81319 (17)	−0.1596 (2)	0.59275 (14)	0.0523 (5)	
H10	0.8165	−0.1042	0.6518	0.063*	
C11	0.89636 (15)	−0.2462 (2)	0.58046 (15)	0.0524 (5)	
H11	0.9593	−0.2491	0.6308	0.063*	
C12	0.89261 (13)	−0.3286 (2)	0.49875 (13)	0.0425 (4)	
H12	0.9522	−0.3885	0.4914	0.051*	
C13	0.80189 (12)	−0.32480 (16)	0.42671 (11)	0.0315 (3)	
C14	0.62180 (11)	−0.22199 (15)	0.35457 (10)	0.0270 (3)	
C15	0.62678 (17)	−0.0059 (2)	0.61327 (15)	0.0523 (5)	
H15A	0.6294 (13)	−0.0783 (10)	0.6634 (8)	0.078*	
H15B	0.5577 (11)	0.0421 (16)	0.6066 (3)	0.078*	
H15C	0.6849 (11)	0.0606 (15)	0.6329 (7)	0.078*	
C16	0.86484 (16)	−0.5108 (2)	0.33788 (14)	0.0477 (5)	
H16A	0.9338 (10)	−0.4683 (6)	0.3302 (11)	0.071*	
H16B	0.8399 (7)	−0.5704 (12)	0.2808 (10)	0.071*	
H16C	0.8742 (9)	−0.5669 (13)	0.3984 (9)	0.071*	

### Atomic displacement parameters (Å^2^)

**Table:**

	*U*^11^	*U*^22^	*U*^33^	*U*^12^	*U*^13^	*U*^23^
O5	0.0399 (7)	0.0729 (10)	0.0426 (7)	−0.0057 (7)	−0.0136 (6)	−0.0001 (7)
N1	0.0484 (8)	0.0244 (7)	0.0428 (8)	0.0029 (6)	−0.0181 (7)	0.0010 (6)
N2	0.0499 (9)	0.0259 (7)	0.0573 (10)	0.0001 (6)	−0.0121 (8)	0.0043 (7)
C1	0.0278 (7)	0.0275 (7)	0.0339 (7)	0.0028 (6)	−0.0002 (6)	−0.0054 (6)
C2	0.0279 (7)	0.0432 (9)	0.0391 (9)	−0.0068 (7)	−0.0025 (6)	0.0002 (7)
C3	0.0344 (8)	0.0496 (10)	0.0363 (8)	−0.0056 (7)	−0.0002 (6)	0.0046 (7)
C4	0.0280 (7)	0.0481 (10)	0.0340 (8)	0.0026 (7)	−0.0022 (6)	−0.0077 (7)
C5	0.0310 (8)	0.0456 (10)	0.0478 (10)	−0.0105 (7)	−0.0042 (7)	−0.0070 (8)
C6	0.0371 (8)	0.0335 (8)	0.0440 (9)	−0.0063 (7)	0.0005 (7)	−0.0015 (7)
C7	0.0307 (7)	0.0247 (7)	0.0372 (8)	0.0049 (6)	−0.0013 (6)	−0.0019 (6)
C17	0.0533 (12)	0.0791 (16)	0.0397 (10)	0.0039 (11)	−0.0081 (8)	0.0063 (10)
O1	0.0410 (6)	0.0318 (6)	0.0416 (6)	−0.0013 (5)	−0.0104 (5)	0.0114 (5)
O2	0.0363 (6)	0.0267 (6)	0.0568 (8)	−0.0032 (5)	−0.0126 (5)	0.0046 (5)
O3	0.0525 (8)	0.0551 (8)	0.0425 (7)	0.0201 (6)	−0.0072 (6)	−0.0171 (6)
O4	0.0446 (7)	0.0508 (7)	0.0354 (6)	0.0217 (6)	−0.0034 (5)	−0.0049 (5)
C8	0.0273 (7)	0.0251 (7)	0.0279 (7)	−0.0004 (5)	−0.0027 (5)	0.0048 (5)
C9	0.0378 (8)	0.0331 (8)	0.0356 (8)	0.0037 (6)	−0.0043 (6)	−0.0027 (6)
C10	0.0547 (11)	0.0540 (12)	0.0405 (10)	0.0065 (9)	−0.0154 (8)	−0.0128 (9)
C11	0.0418 (10)	0.0579 (12)	0.0486 (11)	0.0046 (9)	−0.0204 (8)	−0.0008 (9)
C12	0.0302 (8)	0.0475 (10)	0.0459 (10)	0.0094 (7)	−0.0052 (7)	0.0060 (8)
C13	0.0310 (7)	0.0326 (8)	0.0295 (7)	0.0035 (6)	−0.0001 (6)	0.0054 (6)
C14	0.0272 (7)	0.0237 (7)	0.0281 (7)	0.0064 (5)	−0.0018 (5)	−0.0015 (5)
C15	0.0532 (11)	0.0604 (12)	0.0452 (10)	0.0034 (9)	0.0133 (9)	−0.0112 (9)
C16	0.0494 (10)	0.0501 (11)	0.0445 (10)	0.0213 (9)	0.0103 (8)	0.0032 (8)

### Geometric parameters (Å, °)

**Table:**

O5—C4	1.3680 (19)	O1—C14	1.2509 (18)
O5—C17	1.427 (3)	O2—C14	1.2440 (18)
N1—C7	1.299 (2)	O3—C9	1.368 (2)
N1—H1A	0.93 (2)	O3—C15	1.427 (2)
N1—H1B	0.88 (2)	O4—C13	1.370 (2)
N2—C7	1.316 (2)	O4—C16	1.418 (2)
N2—H2A	0.91 (3)	C8—C9	1.391 (2)
N2—H2B	0.86 (3)	C8—C13	1.392 (2)
C1—C2	1.387 (2)	C8—C14	1.5189 (18)
C1—C6	1.394 (2)	C9—C10	1.394 (2)
C1—C7	1.480 (2)	C10—C11	1.377 (3)
C2—C3	1.386 (2)	C10—H10	0.9700
C2—H2	0.9700	C11—C12	1.375 (3)
C3—C4	1.386 (2)	C11—H11	0.9700
C3—H3	0.9700	C12—C13	1.393 (2)
C4—C5	1.385 (3)	C12—H12	0.9700
C5—C6	1.383 (2)	C15—H15A	0.9817
C5—H5	0.9700	C15—H15B	0.9817
C6—H6	0.9700	C15—H15C	0.9817
C17—H17A	0.9877	C16—H16A	0.9865
C17—H17B	0.9877	C16—H16B	0.9865
C17—H17C	0.9877	C16—H16C	0.9865
			
C4—O5—C17	117.56 (15)	C13—O4—C16	117.61 (13)
C7—N1—H1A	120.8 (13)	C9—C8—C13	119.07 (13)
C7—N1—H1B	122.1 (14)	C9—C8—C14	119.47 (13)
H1A—N1—H1B	116.8 (19)	C13—C8—C14	121.43 (13)
C7—N2—H2A	117.3 (16)	O3—C9—C8	115.43 (13)
C7—N2—H2B	120.8 (16)	O3—C9—C10	123.91 (16)
H2A—N2—H2B	121 (2)	C8—C9—C10	120.65 (16)
C2—C1—C6	118.52 (14)	C11—C10—C9	118.85 (17)
C2—C1—C7	119.69 (14)	C11—C10—H10	120.6
C6—C1—C7	121.78 (15)	C9—C10—H10	120.6
C3—C2—C1	121.67 (15)	C12—C11—C10	121.83 (16)
C3—C2—H2	119.2	C12—C11—H11	119.1
C1—C2—H2	119.2	C10—C11—H11	119.1
C4—C3—C2	118.91 (16)	C11—C12—C13	119.02 (16)
C4—C3—H3	120.5	C11—C12—H12	120.5
C2—C3—H3	120.5	C13—C12—H12	120.5
O5—C4—C5	115.80 (15)	O4—C13—C8	115.57 (13)
O5—C4—C3	123.89 (17)	O4—C13—C12	123.92 (15)
C5—C4—C3	120.31 (15)	C8—C13—C12	120.52 (15)
C6—C5—C4	120.24 (15)	O2—C14—O1	125.56 (13)
C6—C5—H5	119.9	O2—C14—C8	118.88 (13)
C4—C5—H5	119.9	O1—C14—C8	115.54 (13)
C5—C6—C1	120.34 (16)	O3—C15—H15A	109.5
C5—C6—H6	119.8	O3—C15—H15B	109.5
C1—C6—H6	119.8	H15A—C15—H15B	109.5
N1—C7—N2	119.47 (15)	O3—C15—H15C	109.5
N1—C7—C1	119.60 (14)	H15A—C15—H15C	109.5
N2—C7—C1	120.93 (15)	H15B—C15—H15C	109.5
O5—C17—H17A	109.5	O4—C16—H16A	109.5
O5—C17—H17B	109.5	O4—C16—H16B	109.5
H17A—C17—H17B	109.5	H16A—C16—H16B	109.5
O5—C17—H17C	109.5	O4—C16—H16C	109.5
H17A—C17—H17C	109.5	H16A—C16—H16C	109.5
H17B—C17—H17C	109.5	H16B—C16—H16C	109.5
C9—O3—C15	118.21 (14)		
			
C6—C1—C2—C3	−0.2 (3)	C14—C8—C9—O3	1.5 (2)
C7—C1—C2—C3	−179.38 (16)	C13—C8—C9—C10	0.1 (3)
C1—C2—C3—C4	0.9 (3)	C14—C8—C9—C10	−177.89 (17)
C17—O5—C4—C5	172.81 (18)	O3—C9—C10—C11	−177.5 (2)
C17—O5—C4—C3	−7.5 (3)	C8—C9—C10—C11	1.9 (3)
C2—C3—C4—O5	179.46 (17)	C9—C10—C11—C12	−1.8 (3)
C2—C3—C4—C5	−0.8 (3)	C10—C11—C12—C13	−0.3 (3)
O5—C4—C5—C6	179.86 (17)	C16—O4—C13—C8	−172.11 (15)
C3—C4—C5—C6	0.1 (3)	C16—O4—C13—C12	8.1 (3)
C4—C5—C6—C1	0.6 (3)	C9—C8—C13—O4	177.88 (15)
C2—C1—C6—C5	−0.5 (3)	C14—C8—C13—O4	−4.2 (2)
C7—C1—C6—C5	178.64 (16)	C9—C8—C13—C12	−2.3 (2)
C2—C1—C7—N1	27.6 (2)	C14—C8—C13—C12	175.68 (15)
C6—C1—C7—N1	−151.54 (17)	C11—C12—C13—O4	−177.78 (17)
C2—C1—C7—N2	−152.93 (18)	C11—C12—C13—C8	2.4 (3)
C6—C1—C7—N2	27.9 (2)	C9—C8—C14—O2	−107.53 (17)
C15—O3—C9—C8	164.66 (17)	C13—C8—C14—O2	74.5 (2)
C15—O3—C9—C10	−15.9 (3)	C9—C8—C14—O1	71.0 (2)
C13—C8—C9—O3	179.53 (15)	C13—C8—C14—O1	−106.91 (17)

### Hydrogen-bond geometry (Å, °)

**Table:**

*D*—H···*A*	*D*—H	H···*A*	*D*···*A*	*D*—H···*A*
N1—H1A···O1	0.93 (2)	1.84 (2)	2.7576 (18)	169 (2)
N1—H1B···O2^i^	0.88 (2)	1.91 (2)	2.7166 (18)	152 (2)
N2—H2B···O1^ii^	0.86 (3)	2.31 (2)	2.868 (2)	123 (2)
N2—H2A···O2	0.91 (3)	2.08 (3)	2.976 (2)	170 (2)

Symmetry codes: (i) −*x*+1, *y*+1/2, −*z*+1/2; (ii) −*x*+1, *y*−1/2, −*z*+1/2.
